# Supplementary material for: Association of Differences in Dietary Behaviours and Lifestyle with Self-Reported Weight Gain during the COVID-19 Lockdown in a University Community from Chile: A Cross-Sectional Study
Source: Nutrients. 2021 Sep 16;13(9):3213. doi: 10.3390/nu13093213 (PMC8471452; doi:10.3390/nu13093213)
Supplement: Supplementary file 1 [file nutrients-13-03213-s001.zip › nutrients-1362109-supplementary.pdf]

SUPPLEMENTARY DATA

Supplementary Table S1. Descriptive characteristics of the population, disaggregated by weight gain during lockdown.									
	Total n=639		No weight gain n=407		5-9% weight gain n=164		≥10% weight gain n=68		
	Mean	Standard deviation	Mean	Standard deviation	Mean	Standard deviation	Mean	Standard deviation	p value
**Age, years**	28.9	13.2	29.9	13.9	28.3	12.3	25.4*	8.8	0.024
**BMI before lockdown**	25.1	4.8	25.2	5.2	24.9	3.9	24.9	4.4	0.719
**BMI during lockdown**	25.8	5.0	25.1	4.2	26.6*	4.2	28.3*	5.1	<0.001
Sociodemographic and feeding behaviour data									
	Frecuency	Percentage	Frecuency	Percentage	Frecuency	Percentage	Frecuency	Percentage	
**Place of residence**									
Urban	546	85.4	348	85.5	141	86.0	57	83.8	0.825
Rural	93	14.6	59	14.5	23	14.0	11	16.2	
**Occupation**									
Student	461	72.1	282	69.3	123	75.0	56	82.4	0.119
Academic	84	13.1	67	16.5	12	7.3	5	7.4	
Administrative	94	14.7	58	14.3	29	17.7	7	10.3	
**Educational level**									
Basic (incomplete)	4	0.6	3	0.7	1	0.6	0	0.0	0.028
Basic (complete)	30	4.7	21	5.2	6	3.7	3	4.4	
Technical (incomplete)	6	0.9	2	0.5	1	0.6	3	4.4	
Technical (complete)	26	4.1	14	3.4	9	5.5	3	4.4	
University (incomplete)	405	63.4	243	59.7	118	72.0	44	64.7	
University (complete)	71	11.1	47	11.5	13	7.9	11	16.2	

Postgraduate	97	15.2	77	18.9	16	9.8	4	5.9	
**Telecommuting from home**	622	97.3	395	97.1	161	98.2	66	97.1	0.723
**Time for sedentary activities**									
One to two hours a day	32	5.0	24	5.9	8	4.9	0	0.0	0.010
Three to four hours a day	70	11.0	47	11.5	15	9.1	8	11.8	
Five to six hours a day	109	17.1	76	18.7	23	14.0	10	14.7	
Seven to eight hours	161	25.2	103	25.3	40	24.4	18	26.5	
Nine o ten hours a day	108	16.9	68	16.7	29	17.7	11	16.2	
Ten or more hours a day	159	24.9	89	21.9	49	29.9	21	30.9	
**Household members**									
Lives alone	28	4.4	21	5.2	6	3.7	1	1.5	0.746
Lives with family (parents and/or siblings or partner and children)	529	82.8	333	81.8	141	86.0	55	80.9	
Lives with relatives who are not parents and siblings	28	4.4	17	4.2	6	3.7	5	7.4	
Lives with friends	7	1.1	5	1.2	1	0.6	1	1.5	
Lives with other people who are not family members	9	1.4	5	1.2	2	1.2	2	2.9	
Lives with partner	35	5.5	24	5.9	8	4.9	3	4.4	
Other	3	0.5	2	0.5	0	0.0	1	1.5	
**Lunch**	365	57.1	237	58.2	95	57.9	33	48.5	0.223

**Away from home**	297	46.5	190	46.7	80	48.8	27	39.7	0.526
**Packed lunch for work**	206	32.2	127	31.2	58	35.4	21	30.9	0.683
**Differences in habits**									
Yes, it has gotten worse	268	41.9	112	27.5	106	64.6	50	73.5	<0.001
Yes, it has improved	190	29.7	152	37.3	27	16.5	11	16.2	
No, it has stayed the same	181	28.3	143	35.1	31	18.9	7	10.3	
**Mealtimes before the pandemic**									
Breakfast	521	81.5	326	80.1	135	82.3	60	88.2	0.119
Mid-morning snack	263	41.2	174	42.8	65	39.6	24	35.3	0.216
Lunch	613	95.9	394	96.8	156	95.1	63	92.6	0.088
Mid-afternoon snack	291	45.5	187	45.9	76	46.3	28	41.2	0.590
Snack before dinner	517	80.9	317	77.9	140	85.4	60	88.2	0.011
Dinner	141	22.1	100	24.6	34	20.7	7	10.3	0.011
Late-night snack	119	18.6	84	20.6	23	14.0	12	17.6	0.185
Snacking between meals	219	34.3	144	35.4	51	31.1	24	35.3	0.640
**Mealtimes during the pandemic**									
Breakfast	504	78.9	328	80.6	126	76.8	50	73.5	0.130
Mid-morning snack	164	25.7	98	24.1	49	29.9	17	25.0	0.424
Lunch	623	97.5	395	97.1	162	98.8	66	97.1	0.574
Mid-afternoon snack	294	46.0	181	44.5	79	48.2	34	50.0	0.295
Snack before dinner	529	82.8	322	79.1	147	89.6	60	88.2	0.004
Dinner	163	25.5	114	28.0	34	20.7	15	22.1	0.095
Late-night snack	214	33.5	117	28.7	66	40.2	31	45.6	0.001

Snacking between meals	319	49.9	157	38.6	114	69.5	48	70.6	<0.001
**Snack between meals**									
Does not snack between meals	95	14.9	83	20.4	9	5.5	3	4.4	0.005
Same as before	120	18.8	88	21.6	23	14.0	9	13.2	
More than before	282	44.1	125	30.7	109	66.5	48	70.6	
Less than before	142	22.2	111	27.3	23	14.0	8	11.8	
**Emotional feeding behaviour**									
No emotional feeding behaviour	121	18.9	99	24.3	18	11.0	4	5.9	<0.001
Little emotional feeding behaviour	204	31.9	146	35.9	43	26.2	15	22.1	
Some emotional feeding behaviour	260	40.7	140	34.4	85	51.8	35	51.5	
Emotional feeding behaviour	54	8.5	22	5.4	18	11.0	14	20.6	
**Lifestyle before the pandemic**									
Fantastic lifestyle	76	11.9	48	11.8	20	12.2	8	11.8	0.229
Right path	306	47.9	203	49.9	74	45.1	29	42.6	
Adequate	164	25.7	102	25.1	44	26.8	18	26.5	
Could be better	89	13.9	51	12.5	25	15.2	13	19.1	
Danger zone	4	0.6	3	0.7	1	0.6	0	0.0	
**Lifestyle during the pandemic**									
Fantastic lifestyle	26	4.1	24	5.9	2	1.2	0	0.0	<0.001

Right path	177	27.7	134	32.9	33	20.1	10	14.7	
Adequate	167	26.1	109	26.8	38	23.2	20	29.4	
Could be better	242	37.9	135	33.2	81	49.4	26	38.2	
Danger zone	27	4.2	5	1.2	10	6.1	12	17.6	
**Food Safety**									
Safe	222	34.7	158	38.8	47	28.7	17	25.0	0.001
Mildly unsafe	265	41.5	163	40.0	71	43.3	31	45.6	
Moderately unsafe	86	13.5	51	12.5	29	17.7	6	8.8	
Severely unsafe	66	10.3	35	8.6	17	10.4	14	20.6	

Comparisons were made with ANOVA one-way and Dunnett poshoc test, X^2, and X^2 for trend

*statistically significant difference with respect to the group of No weight gain.

Supplementary Table S2. Descriptive data of the frequencies of self-reported food intake before and during the lockdown					
	Before lockdown		During lockdown		
	Frequency	Percentage	Frequency	Percentage	Valor de p
**White or whole wheat bread**					0.013
Never	85	13.3	83	13.0	
Once a month or less	63	9.9	60	9.4	
Once a week or every 15 days	54	8.5	45	7.0	
2-3 times a week	94	14.7	90	14.1	
4-6 times a week	59	9.2	90	14.1	
Everyday	216	33.8	189	29.6	
Every day, several times a day	68	10.6	82	12.8	
**Rice, potatoes, noodles, or quinoa**					<0.001
Never	7	1.1	5	0.8	
Once a month or less	18	2.8	8	1.3	
Once a week or every 15 days	100	15.6	71	11.1	
2-3 times a week	322	50.4	271	42.4	
4-6 times a week	139	21.8	220	34.4	
Everyday	50	7.8	57	8.9	
Every day, several times a day	3	0.5	7	1.1	
**Raw and / or cooked vegetables**					0.345
Never	6	0.9	6	0.9	
Once a month or less	17	2.7	16	2.5	
Once a week or every 15 days	73	11.4	63	9.9	
2-3 times a week	174	27.2	151	23.6	
4-6 times a week	132	20.7	156	24.4	
Everyday	190	29.7	198	31.0	
Every day, several times a day	47	7.4	49	7.7	
**Natural fruit (excludes juices)**					0.030
Never	17	2.7	24	3.8	
Once a month or less	43	6.7	52	8.1	
Once a week or every 15 days	129	20.2	120	18.8	
2-3 times a week	200	31.3	173	27.1	
4-6 times a week	115	18.0	116	18.2	

Everyday	87	13.6	111	17.4	
Every day, several times a day	48	7.5	43	6.7	
**Dried vegetables**					<0.001
Never	24	3.8	16	2.5	
Once a month or less	60	9.4	52	8.1	
Once a week or every 15 days	273	42.7	211	33.0	
2-3 times a week	224	35.1	275	43.0	
4-6 times a week	48	7.5	71	11.1	
Everyday	9	1.4	12	1.9	
Every day, several times a day	1	0.2	2	0.3	
**Milk, yogurt, or kefir**					0.518
Never	46	7.2	46	7.2	
Once a month or less	45	7.0	58	9.1	
Once a week or every 15 days	108	16.9	97	15.2	
2-3 times a week	168	26.3	174	27.2	
4-6 times a week	121	18.9	109	17.1	
Everyday	116	18.2	120	18.8	
Every day, several times a day	35	5.5	35	5.5	
**Cheeses (aged, fresh, farm, etc.)**					0.185
Never	40	6.3	48	7.5	
Once a month or less	60	9.4	78	12.2	
Once a week or every 15 days	128	20.0	117	18.3	
2-3 times a week	226	35.4	216	33.8	
4-6 times a week	138	21.6	124	19.4	
Everyday	38	5.9	41	6.4	
Every day, several times a day	9	1.4	15	2.3	
**Meat (pork, chicken, beef, lamb, etc.)**					0.309
Never	46	7.2	53	8.3	
Once a month or less	20	3.1	26	4.1	
Once a week or every 15 days	111	17.4	116	18.2	
2-3 times a week	274	42.9	267	41.8	
4-6 times a week	151	23.6	139	21.8	
Everyday	32	5.0	33	5.2	
Every day, several times a day	5	0.8	5	0.8	
**Processed meats and sausages (Turkey ham,**					0.019

**Viennese, hamburgers, sausage, salami, mortadella, etc.**					
Never	81	12.7	85	13.3	
Once a month or less	77	12.1	94	14.7	
Once a week or every 15 days	157	24.6	178	27.9	
2-3 times a week	186	29.1	167	26.1	
4-6 times a week	111	17.4	87	13.6	
Everyday	25	3.9	25	3.9	
Every day, several times a day	2	0.3	3	0.5	
**Fresh and canned seafood**					0.043
Never	57	8.9	68	10.6	
Once a month or less	135	21.1	112	17.5	
Once a week or every 15 days	267	41.8	256	40.1	
2-3 times a week	156	24.4	168	26.3	
4-6 times a week	19	3.0	33	5.2	
Everyday	5	0.8	2	0.3	
Every day, several times a day	0	0.0	0	0.0	
**Eggs**					0.024
Never	25	3.9	29	4.5	
Once a month or less	23	3.6	32	5.0	
Once a week or every 15 days	106	16.6	106	16.6	
2-3 times a week	268	41.9	239	37.4	
4-6 times a week	142	22.2	162	25.4	
Everyday	62	9.7	57	8.9	
Every day, several times a day	13	2.0	14	2.2	
**Nuts (excludes raisins)**					<0.001
Never	73	11.4	121	18.9	
Once a month or less	142	22.2	154	24.1	
Once a week or every 15 days	187	29.3	157	24.6	
2-3 times a week	125	19.6	111	17.4	
4-6 times a week	63	9.9	58	9.1	
Everyday	34	5.3	28	4.4	
Every day, several times a day	15	2.3	10	1.6	
**Butter, margarine, vegetable oil or fats of animal origin**					0.175
Never	32	5.0	38	5.9	

Once a month or less	60	9.4	75	11.7	
Once a week or every 15 days	109	17.1	98	15.3	
2-3 times a week	162	25.4	159	24.9	
4-6 times a week	123	19.2	121	18.9	
Everyday	125	19.6	120	18.8	
Every day, several times a day	28	4.4	28	4.4	
**Sweet or filled cookies, cakes, etc.**					0.038
Never	23	3.6	37	5.8	
Once a month or less	90	14.1	115	18.0	
Once a week or every 15 days	189	29.6	163	25.5	
2-3 times a week	183	28.6	180	28.2	
4-6 times a week	102	16.0	103	16.1	
Everyday	45	7.0	36	5.6	
Every day, several times a day	7	1.1	5	0.8	
**Chocolates and chocolate-based products**					0.003
Never	34	5.3	54	8.5	
Once a month or less	157	24.6	169	26.4	
Once a week or every 15 days	204	31.9	177	27.7	
2-3 times a week	168	26.3	145	22.7	
4-6 times a week	53	8.3	75	11.7	
Everyday	19	3.0	12	1.9	
Every day, several times a day	4	0.6	7	1.1	
**Salty snacks like French fries, doritos, cheetos, etc.**					0.348
Never	63	9.9	82	12.8	
Once a month or less	150	23.5	155	24.3	
Once a week or every 15 days	229	35.8	191	29.9	
2-3 times a week	131	20.5	142	22.2	
4-6 times a week	49	7.7	50	7.8	
Everyday	12	1.9	16	2.5	
Every day, several times a day	5	0.8	3	0.5	
**Drinks or juices whit added sugar**					0.001
Never	121	18.9	151	23.6	
Once a month or less	113	17.7	141	22.1	

Once a week or every 15 days	137	21.4	113	17.7	
2-3 times a week	131	20.5	117	18.3	
4-6 times a week	76	11.9	60	9.4	
Everyday	43	6.7	39	6.1	
Every day, several times a day	18	2.8	18	2.8	
**Alcoholic drinks**					<0.001
Never	196	30.7	272	42.6	
Once a month or less	194	30.4	175	27.4	
Once a week or every 15 days	159	24.9	105	16.4	
2-3 times a week	74	11.6	57	8.9	
4-6 times a week	8	1.3	17	2.7	
Everyday	7	1.1	11	1.7	
Every day, several times a day	1	0.2	2	0.3	
**Sugar (white or brown)**					0.348
Never	155	24.3	178	27.9	
Once a month or less	83	13.0	78	12.2	
Once a week or every 15 days	63	9.9	67	10.5	
2-3 times a week	74	11.6	64	10.0	
4-6 times a week	47	7.4	41	6.4	
Everyday	139	21.8	130	20.3	
Every day, several times a day	78	12.2	81	12.7	
Data compared by the McNemar test.					

Supplementary Table S3. Univariate results of the linear regression models for the prediction of self-reported weight, BMI, and percentage weight gain during lockdown from higher food intake during lockdown									
	Difference in self-reported weight			BMI difference			Percentage of self-reported weight change		
	B_0	B_1	P	B_0	B_1	P	B_0	B_1	P
White or whole wheat bread	1.69	1.24	0.002	0.61	0.47	<0.001	2.62	1.66	0.002
Rice, potatoes, noodles, or quinoa	1.59	1.27	0.001	0.58	0.48	<0.001	2.45	1.80	0.003
Raw and / or cooked vegetables	2.34	-1.41	<0.001	0.86	-0.51	<0.001	3.43	-1.67	0.002
Natural fruit (excludes juices)	2.33	-1.19	0.002	0.86	-0.44	<0.001	3.48	-1.61	0.002
Dried vegetables	2.14	-0.46	0.201	0.78	-0.16	0.206	3.22	-0.60	0.204
Milk, yogurt, or kefir	2.18	-0.72	0.060	0.80	-0.27	0.064	3.27	-0.93	0.082
Cheeses (aged, fresh, farm, etc.)	1.80	0.87	0.030	0.66	0.33	0.032	2.73	1.32	0.022
Meat (pork, chicken, beef, lamb, etc.)	1.85	0.82	0.070	0.68	0.29	0.073	2.78	1.37	0.031
Processed meats and sausages	1.65	1.77	<0.001	0.60	0.66	<0.001	2.56	2.43	<0.001
Fresh and canned seafood	2.08	-0.31	0.402	0.76	-0.12	0.410	3.10	-0.27	0.600
Eggs	1.98	0.07	0.910	0.73	0.02	0.911	2.97	0.23	0.713
Nuts (excludes raisins)	2.08	-0.43	0.301	0.76	-0.13	0.420	3.11	-0.41	0.502
Butter, margarine, vegetable oil or fats of animal origin	1.77	0.87	0.021	0.65	0.32	0.030	2.73	1.15	0.031
Sweet or filled cookies, cakes, etc.	1.34	2.26	<0.001	0.49	0.84	<0.001	2.08	3.26	<0.001
Chocolates and chocolate-based products	1.47	1.96	<0.001	0.54	0.73	<0.001	2.24	2.93	<0.001
Salty snacks	1.37	2.23	<0.001	0.50	0.83	<0.001	2.15	3.08	<0.001
Drinks or juices whit added sugar	1.58	1.91	<0.001	0.57	0.73	<0.001	2.40	2.87	<0.001
Alcoholic drinks	1.88	0.73	0.101	0.69	0.28	0.094	2.84	1.14	0.080
Sugar (white or brown)	1.69	1.69	<0.001	0.61	0.65	0.001	2.63	2.18	0.003
B_0: Regression constant, B_1: Coefficient									

Supplementary Table S4. Multilevel logistic regression models to determine the odds of a ≥5% self-reported weight gain during lockdown by the consumption frequencies				
		95%CI		
	OR	lower	upper	P value
**White or whole wheat bread**				
Never	Reference			
Once a month or less	0.85	0.43	1.68	0.701
Once a week or every 15 days	0.83	0.40	1.75	0.600
2-3 times a week	0.79	0.43	1.46	0.522
4-6 times a week	0.53	0.28	0.99	0.050
Everyday	0.64	0.37	1.09	0.091
Every day, several times a day	1.51	0.82	2.80	0.211
**Rice, potatoes, noodles, or quinoa**				
Never	Reference			
Once a month or less	0.57	0.03	11.85	0.744
Once a week or every 15 days	1.92	0.20	18.13	0.601
2-3 times a week	1.92	0.21	17.46	0.600
4-6 times a week	3.04	0.33	27.64	0.302
Everyday	2.91	0.31	27.70	0.303
Every day, several times a day	5.33	0.38	75.78	0.203
**Raw and / or cooked vegetables**				
Never	Reference			
Once a month or less	1.00	0.15	6.53	0.905
Once a week or every 15 days	1.10	0.21	5.87	0.909
2-3 times a week	0.54	0.11	2.77	0.407
4-6 times a week	0.49	0.09	2.49	0.302
Everyday	0.62	0.12	3.17	0.504
Every day, several times a day	0.32	0.06	1.83	0.202
**Natural fruit (excludes juices)**				
Never	Reference			
Once a month or less	1.30	0.49	3.44	0.600
Once a week or every 15 days	0.97	0.40	2.35	0.999
2-3 times a week	0.86	0.36	2.06	0.700
4-6 times a week	0.68	0.28	1.68	0.404
Everyday	0.76	0.31	1.87	0.505
Every day, several times a day	0.37	0.12	1.11	0.080
**Dried vegetables**				
Never	Reference			
Once a month or less	1.32	0.42	4.18	0.606
Once a week or every 15 days	0.94	0.33	2.68	0.900
2-3 times a week	1.00	0.35	2.83	0.905
4-6 times a week	0.96	0.31	2.96	0.910
Everyday	0.33	0.05	2.07	0.210
Every day, several times a day	0.00	0.00		0.999
**Milk, yogurt, or kefir**				
Never	Reference			

Once a month or less	1.79	0.81	3.92	0.105
Once a week or every 15 days	1.35	0.66	2.75	0.402
2-3 times a week	0.84	0.43	1.64	0.600
4-6 times a week	0.80	0.39	1.63	0.502
Everyday	0.57	0.28	1.16	0.101
Every day, several times a day	0.81	0.32	2.03	0.604
**Cheeses (aged, fresh, farm, etc.)**				
Never	Reference			
Once a month or less	1.51	0.69	3.31	0.309
Once a week or every 15 days	1.40	0.67	2.94	0.302
2-3 times a week	1.58	0.79	3.17	0.104
4-6 times a week	1.64	0.79	3.42	0.140
Everyday	3.12	1.29	7.55	0.010
Every day, several times a day	1.79	0.53	6.04	0.350
**Meat (pork, chicken, beef, lamb, etc.)**				
Never	Reference			
Once a month or less	1.03	0.39	2.71	0.966
Once a week or every 15 days	0.97	0.50	1.90	0.921
2-3 times a week	0.84	0.46	1.55	0.540
4-6 times a week	1.15	0.60	2.20	0.674
Everyday	1.22	0.50	2.95	0.604
Every day, several times a day	1.10	0.17	7.16	0.909
**Processed meats and sausages (Turkey ham, Viennese, hamburgers, sausage, salami, mortadella, etc.**				
Never	Reference			
Once a month or less	1.10	0.59	2.05	0.700
Once a week or every 15 days	1.01	0.58	1.75	0.902
2-3 times a week	1.23	0.71	2.14	0.401
4-6 times a week	1.99	1.07	3.69	0.030
Everyday	1.15	0.45	2.91	0.730
Every day, several times a day	1.02	0.09	11.71	0.930
**Fresh and canned seafood**				
Never	Reference			
Once a month or less	0.84	0.45	1.55	0.530
Once a week or every 15 days	0.79	0.46	1.37	0.450
2-3 times a week	0.65	0.37	1.17	0.139
4-6 times a week	0.87	0.37	2.04	0.720
Everyday	1.34	0.08	22.41	0.822
Every day, several times a day	0.00	0.00	0.00	<0.001
**Eggs**				
Never	Reference			
Once a month or less	1.25	0.45	3.45	0.614
Once a week or every 15 days	0.79	0.34	1.83	0.543
2-3 times a week	0.70	0.32	1.54	0.329
4-6 times a week	0.79	0.35	1.77	0.538

Everyday	1.57	0.64	3.89	0.334
Every day, several times a day	0.57	0.14	2.24	0.435
**Nuts (excludes raisins)**				
Never	Reference			
Once a month or less	1.07	0.66	1.74	0.719
Once a week or every 15 days	0.88	0.54	1.44	0.629
2-3 times a week	0.62	0.36	1.07	0.084
4-6 times a week	0.56	0.28	1.11	0.090
Everyday	0.70	0.29	1.67	0.400
Every day, several times a day	1.47	0.40	5.35	0.514
**Butter, margarine, vegetable oil or fats of animal origin**				
Never	Reference			
Once a month or less	0.74	0.31	1.74	0.415
Once a week or every 15 days	0.78	0.35	1.77	0.514
2-3 times a week	1.18	0.55	2.51	0.620
4-6 times a week	1.93	0.89	4.17	0.060
Everyday	1.60	0.74	3.47	0.201
Every day, several times a day	2.50	0.91	6.86	0.074
**Sweet or filled cookies, cakes, etc.**				
Never	Reference			
Once a month or less	1.60	0.56	4.56	0.340
Once a week or every 15 days	2.52	0.92	6.86	0.070
2-3 times a week	6.12	2.28	16.42	<0.001
4-6 times a week	5.81	2.10	16.09	0.001
Everyday	8.00	2.54	25.24	<0.001
Every day, several times a day	No estimable			
**Chocolates and chocolate-based products**				
Never	Reference			
Once a month or less	1.14	0.56	2.33	0.740
Once a week or every 15 days	1.70	0.85	3.41	0.141
2-3 times a week	2.63	1.30	5.33	0.007
4-6 times a week	3.42	1.58	7.39	0.002
Everyday	3.15	0.87	11.48	0.100
Every day, several times a day	7.88	1.36	45.58	0.020
**Salty snacks like French fries, doritos, cheetos, etc.**				
Never	Reference			
Once a month or less	1.20	0.62	2.34	0.540
Once a week or every 15 days	2.44	1.31	4.54	0.005
2-3 times a week	4.62	2.44	8.74	<0.001
4-6 times a week	5.25	2.40	11.47	<0.001
Everyday	5.30	1.72	16.40	0.004
Every day, several times a day	8.25	0.70	96.74	0.094
**Drinks or juices whit added sugar**				
Never	Reference			
Once a month or less	0.83	0.49	1.40	0.401

Once a week or every 15 days	1.43	0.85	2.41	0.111
2-3 times a week	2.74	1.65	4.54	<0.001
4-6 times a week	1.92	1.03	3.57	0.034
Everyday	2.93	1.42	6.03	0.004
Every day, several times a day	2.51	0.93	6.75	0.060
**Alcoholic drinks**				
Never	Reference			
Once a month or less	0.98	0.66	1.46	0.991
Once a week or every 15 days	1.03	0.64	1.63	0.923
2-3 times a week	0.83	0.46	1.52	0.505
4-6 times a week	0.91	0.33	2.53	0.844
Everyday	0.63	0.16	2.41	0.413
Every day, several times a day	1.67	0.10	26.94	0.702
**Sugar (white or brown)**				
Never	Reference			
Once a month or less	0.90	0.51	1.59	0.701
Once a week or every 15 days	1.28	0.71	2.29	0.400
2-3 times a week	0.92	0.50	1.69	0.740
4-6 times a week	1.29	0.64	2.60	0.401
Everyday	1.26	0.79	2.02	0.309
Every day, several times a day	2.07	1.21	3.53	0.008

OR: Odds ratio, 95%CI: 95% Confidence interval

Supplementary Figure S1. Difference between self-reported weight and BMI before and during lockdown.

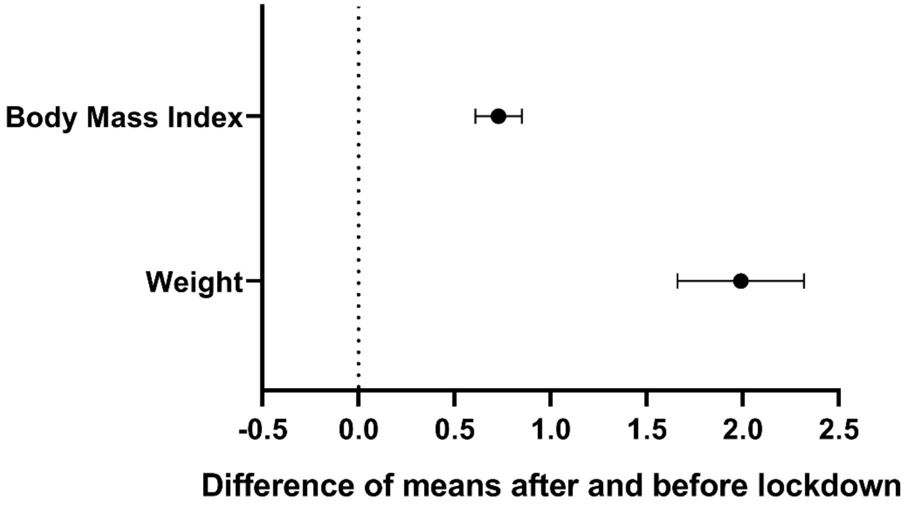

Mean of the difference and 95% CI of the difference are shown.

Supplementary Figure S2. Univariate logistic regression models to determine the odds of ≥5% self-reported weight gain during lockdown from demographic variables.

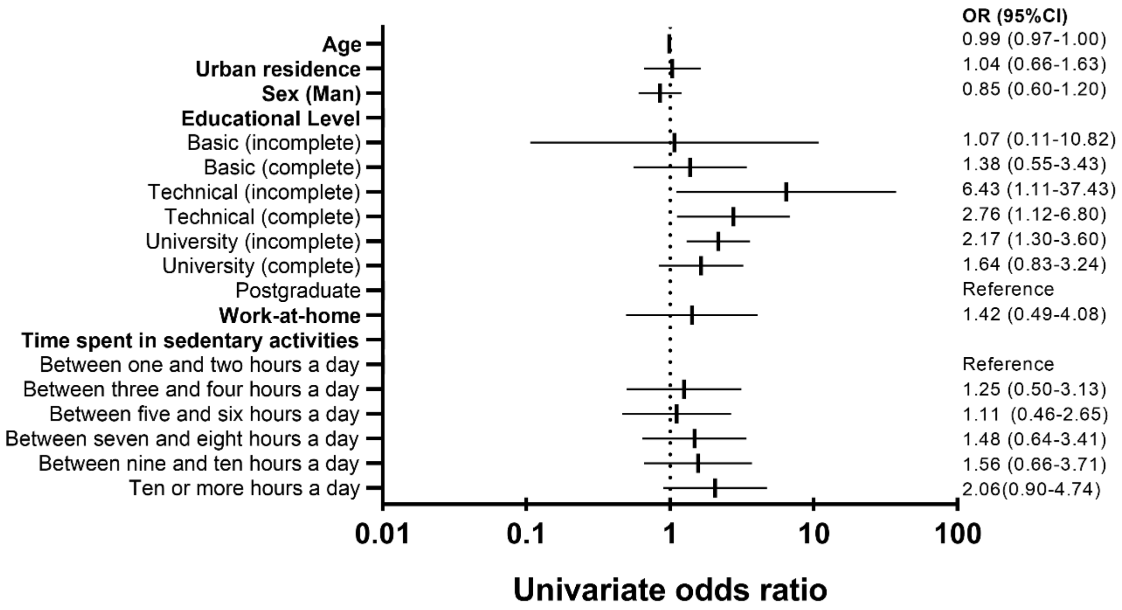

OR: Odds Ratio, 95% CI: 95% Confidence interval

Supplementary Figure S3. Univariate logistic regression models to determine the odds of ≥5% self-reported weight gain from eating habits during lockdown.

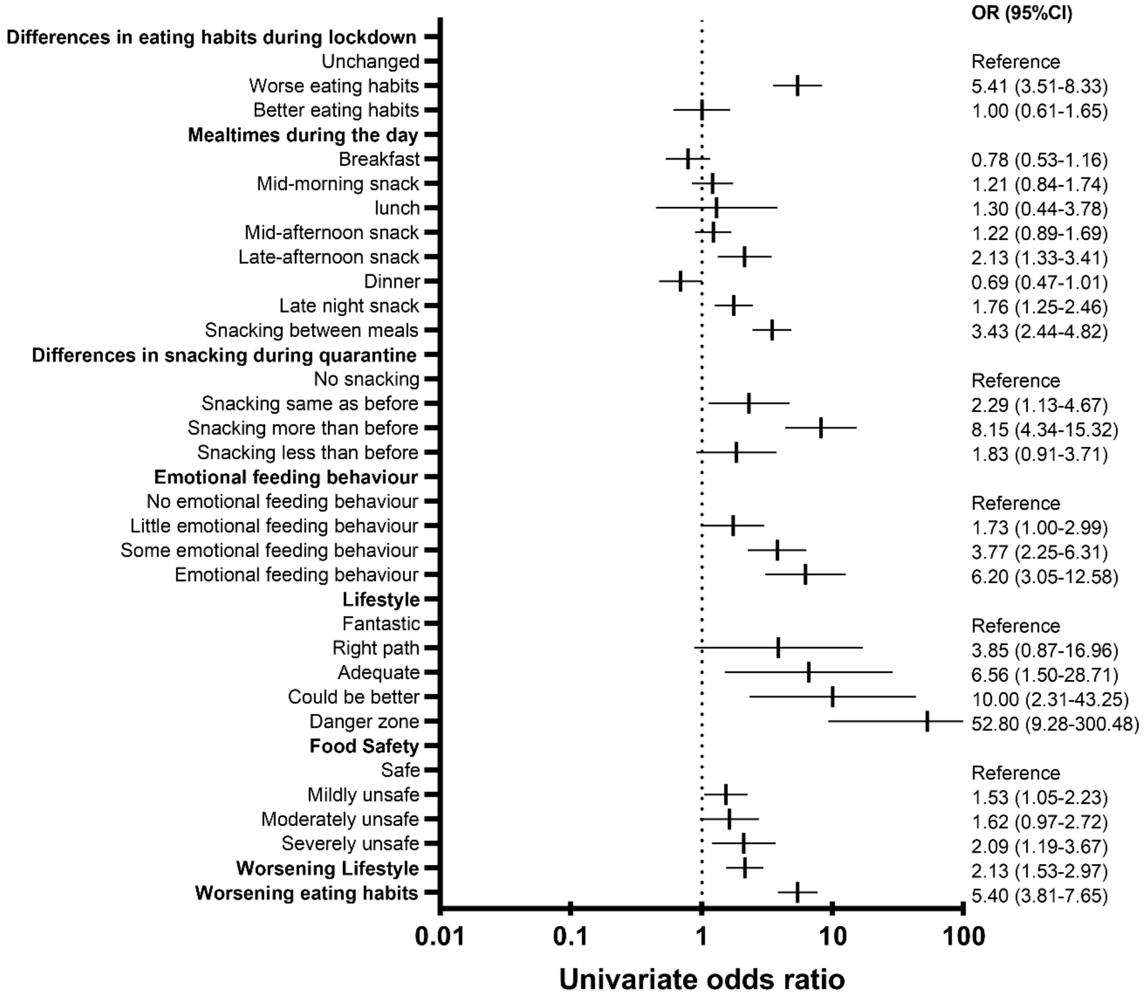

OR: Odds Ratio, 95% CI: 95% Confidence interval

Supplementary Figure S4. Univariate logistic regression models to determine the odds of ≥5% self-reported weight gain during lock-down from more dietary intake during lockdown.

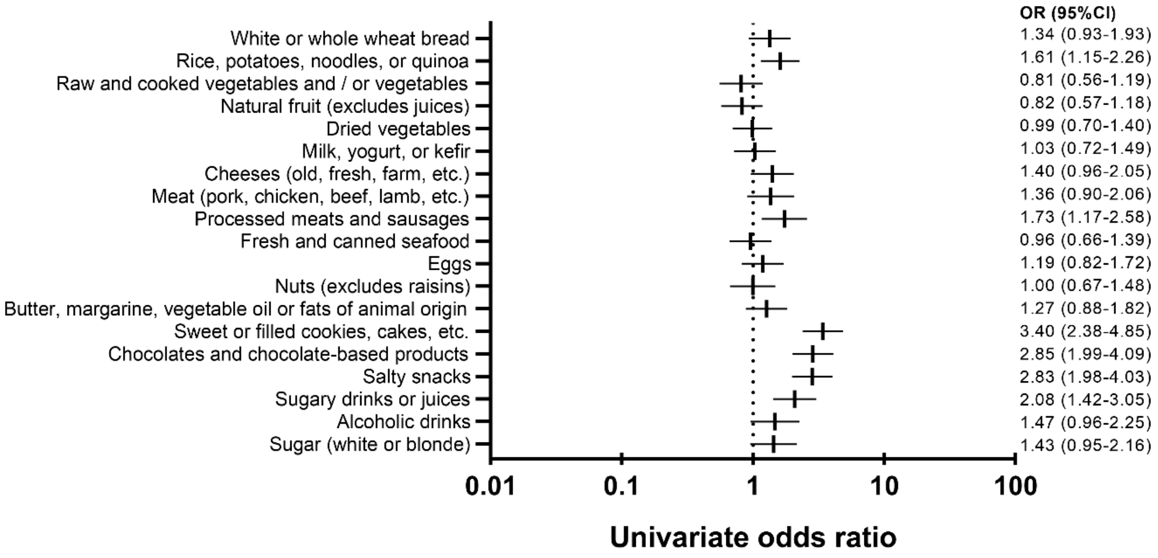

OR: Odds Ratio, 95% CI: 95% Confidence interval
